# Rates of infectious keratitis and other ocular surface adverse events in corneal cross-linking for keratoconus and corneal ectasias performed in an office-based setting: a retrospective cohort study

**DOI:** 10.1186/s40662-023-00354-1

**Published:** 2023-09-01

**Authors:** Farhad Hafezi, Emilio A. Torres-Netto, Leonard Kollros, Nan-Ji Lu, Nikki Hafezi, Cosimo Mazzotta, M. Enes Aydemir, Mark Hillen

**Affiliations:** 1 Laboratory for Ocular Cell Biology, Center for Applied Biotechnology and Molecular Medicine, University of Zurich, Zurich, Switzerland; 2 ELZA Institute, Dietikon, Switzerland; 3 USC Roski Eye Institute, University of Southern California, Los Angeles, CA USA; 4 Faculty of Medicine, University of Geneva, Geneva, Switzerland; 5 Dept. of Ophthalmology, University of Wenzhou, Wenzhou, China; 6 Departmental Ophthalmology Unit, Alta Val d’Elsa Hospital, AUSL Tuscany South-East, Siena, Italy; 7 Postgraduate Ophthalmology School, University of Siena, Siena, Italy; 8 Siena Crosslinking Center, Monteriggioni, Siena, Italy

**Keywords:** Corneal cross-linking, Office-based, Keratoconus, Cornea, Slit lamp, Epithelium-off, Infectious keratitis, Sterile infiltrates

## Abstract

**Background:**

This study aimed to compare the complication rates of epithelium-off corneal cross-linking (epi-off CXL) performed in an office-based setting with those of epi-off CXL performed in an operating room.

**Methods:**

A retrospective cohort study, comprising 501 consecutive epi-off CXL procedures, performed in a non-sterile procedure room without laminar flow ventilation at the ELZA Institute in Zurich, Switzerland, between November 2015 and October 2021, was conducted.

**Results:**

No cases of postoperative infectious keratitis were observed, while sterile infiltrates occurred in 10 out of 501 (2.00%) patients, all of whom responded well to topical steroid therapy. Delayed epithelialization (> 7 days) occurred in 14 out of 501 (2.79%) patients. No other adverse events were noted.

**Conclusions:**

Office-based epi-off CXL does not appear to be associated with an increased risk of complications when compared to operating room settings.

**Supplementary Information:**

The online version contains supplementary material available at 10.1186/s40662-023-00354-1.

## Background

Corneal cross-linking (CXL) is a surgical procedure commonly performed to halt the progression of corneal ectasias like keratoconus or postoperative ectasia [1]. CXL requires the corneal stroma to be saturated with riboflavin, which is then irradiated with ultraviolet (UV)-A light. This reaction results in the photochemical activation of riboflavin and the generation of reactive oxygen species (ROS), which covalently cross-link stromal molecules (predominantly collagen, but also proteoglycans), which renders a stiffer, biomechanically stronger cornea more resistant to ectasia progression. The CXL procedure typically involves epithelial cell debridement of the central 7–9 mm of the cornea (known as “epi-off” CXL), since riboflavin is a large molecule (376.36 g/mol) and does not pass through the epithelium easily [1, 2]. Once UV irradiation is complete, epithelial cells repopulate the corneal surface over the next few days; during this period, patients require topical antimicrobial and pain management.

Despite the associated costs and administrative burden, CXL is typically performed in an operating room, as the sterile environment is considered to be a safe setting for performing epithelial debridement, riboflavin instillation, and the irradiation of a cornea with a large epithelial defect for a period of up to 30 min [3]. However, the ROS generated by the UV-riboflavin photochemical reaction also reduce the microbial load on the cornea to such an extent that riboflavin/UV CXL can be used to treat cases of bacterial, fungal, or mixed bacterial/fungal infections of the cornea [4] in a procedure called photoactivated chromophore for keratitis-CXL (PACK-CXL) [5]. PACK-CXL has been successfully used as an infectious keratitis monotherapy, as well as in combination with antimicrobial pharmacotherapy [5–7]. Considering the cost and administrative burdens of utilizing an operating room [3, 8], its main advantage, sterility, appears to be negated by the antimicrobial effect of CXL/PACK-CXL. The fact that comprehensive antimicrobial prophylaxis is applied after UV irradiation is complete raises the possibility that CXL could be performed in a non-sterile procedure room without an additional infection risk compared to CXL performed in an operating room [3, 6]. This mirrors the transition of intravitreal anti-vascular endothelial growth factor (VEGF) therapies [9–12] and even cataract surgery [13], from the operating theater to doctors’ offices or procedure rooms.

The focus of this paper is narrow: it does not report on the clinical results of CXL for keratoconus, but solely retrospectively examines the adverse event rates of CXL performed in a procedure room over a five-year period in a single office-based non-sterile setting in order to compare these rates with published adverse event rates of CXL performed in an operating room setting.

## Methods

### Surgical technique

#### Patients

This retrospective cohort study involved the analysis of individuals who underwent epi-off CXL procedures for the treatment of corneal ectasia in an office-based, non-sterile setting (a 16 m^2^ procedure room without laminar flow ventilation or humidity control) at the ELZA Institute in Zurich, Switzerland, between November 2015 and October 2021, as previously described [3]. The study was registered with the local ethics committee, the Zurich Kantonale Ethikkommission (ZKE), under the reference REG-2021-01121. As the study was anonymized and retrospectively examined outcomes data only, the ethics committee waived the need for written informed consent and ethical approval for the study. To clarify, all patients had submitted written informed consent to undergo the original surgical procedure.

#### CXL procedure

All patients in this study underwent epi-off CXL. Ten minutes prior to the procedure, patients received topical ocular anesthesia drops: oxybuprocaine (oxybuprocaine hydrochloride, 4 mg/mL, Théa Pharma SA, Clermont-Ferrand, France) followed by one drop of Tetracain (tetracaine 1%, Théa Pharma SA, Clermont-Ferrand, France) every 3 min for a 9-min period. After being brought into the procedure room and the surgical area covered with sterile drapes, the periorbital region was thoroughly disinfected with sterile cotton wool buds soaked in octenidine hydrochloride (Octenisept; Schülke & Mayr GmbH, Norderstedt, Germany). An eyelid speculum was placed, and sterile surgical gauze was secured with surgical tape laterally to the temporal canthus to absorb riboflavin solution run-off. All persons involved wore masks and sterile gloves, and all surfaces encountering the patient were sterile.

Epithelial debridement was performed mechanically either with a hockey blade, an Amoils brush, or with 40% ethanol applied with a sterile cotton swab in a circular tapping manner for around 30–35 s, replaced with a freshly soaked swab and tapped for a further 30–35 s to loosen the epithelium, then wiped away in a circular motion, then rinsed with balanced salt solution (Table 1). This step was performed either with the patient lying supine in a reclining chair (n = 488) or seated upright at a slit lamp (n = 13). Riboflavin instillation was performed every 2 min for 10 min on all patients lying supine in a reclining chair. Over this nearly seven-year period, only two riboflavin solutions were used (Table 1): Ricrolin + (Sooft, Montegiorgio, Italy) was used on the first 331 eyes, then Ribo-Ker (EMAGine AG, Zug, Switzerland; n = 170). Both solutions share hypo-osmolarity and the absence of carriers like dextran or hydroxypropyl methylcellulose (HPMC). The riboflavin used was changed because of availability issues during the COVID-19 pandemic. The UV irradiation protocol used (in terms of irradiation duration and intensity) was dependent on the age of the patient, severity of the ectasia, corneal thickness, and the establishment of newer cross-linking protocols. Irradiation was performed by the cross-linking devices described in Table 1, and the duration of irradiation ranged from 5 to 30 min, intensity ranged from 3 to 18 mW/cm^2^ and fluence ranged from 5.4 to 10 J/cm^2^.

**Table 1:** Baseline demographics and cross-linking procedure parameters used

Parameter	Value
Gender	
Female	152
Male	349
Age (years)	
Mean (SD)	30.7 (12.4)
Minimum, maximum	5.1, 71.7
Operated eye (OD, OS)	255, 246
Preoperative pachymetry (µm)	
Mean (SD)	460.9 (60.3)
Minimum, maximum	152, 596
Ectasia type (patients, n)	
Keratoconus	440
Post-LASIK ectasia	28
Pellucid marginal degeneration	25
Post-radial keratotomy	4
Terrien marginal degeneration	3
Post-PRK ectasia	1
Epithelium removal method (eyes, n)	
Amoils brush	18
Ethanol/cotton swab	150
Hockey knife	333
Epithelial removal location (eyes, n)	
Reclining chair (supine)	488
Slit lamp (sat upright)	13
Riboflavin applied (eyes, n)	
Ricrolin +	298
Ribo-Ker	203
Riboflavin saturation duration (eyes, n)	
10 min	72
20 min	429
UV irradiation duration (mm:ss)	
Mean (SD)	14:56 (07:25)
Minimum, maximum	4:38, 30:00
Mode	10:00
UV irradiation intensity (eyes, n)	
3 mW/cm^2^	173
9 mW/cm^2^	319
18 mW/cm^2^	9
UV irradiation location (eyes, n)	
Operating theater-microscope	453
Slit lamp	48
UV irradiation device (eyes, n)	
C-Eye	88
CXL-365 Vario	413

*SD* = standard deviation; *LASIK* = laser in situ keratomileusis; *PRK* = photorefractive keratectomy; *UV* = ultraviolet; *CXL* = corneal collagen cross-linking

Corneal pachymetry was measured using the SP-1000 (Tomey, Nagoya, Japan) at the thinnest points immediately after riboflavin application and at the end of UV irradiation. After the procedure, the eye was thoroughly irrigated with balanced salt solution (BSS), and topical antibiotics Tobradex (0.1% tobramycin–0.3% dexamethasone, Novartis Pharma, Basel, Switzerland) and Vigamox (moxifloxacin 0.5%; Alcon, Geneva, Switzerland) were administered immediately afterward, and a bandage contact lens (Air Optix Night&Day; Ciba Vision AG) was used to cover the eye. Finally, the speculum was removed. The post-procedural antimicrobial and pain prophylaxis regimen was as previously described [14].

#### Analysis of postoperative infections

After the procedure, we assessed the following parameters: signs of postoperative microbial infection (within the first 14 days), sterile infiltrates (within the first 14 days), and delayed epithelialization (> 7 days), which were observed via slit lamp biomicroscopy.

## Results

A total of 501 patients with corneal ectasia received CXL in an office-based, non-sterile setting between November 2014 and October 2021, with the majority (440/501, 87.82%) having keratoconus as the indication for the procedure. The other indications were post-LASIK ectasia (28/501, 5.59%), pellucid marginal degeneration (25/501, 4.99%), radial keratotomy (4/501, 0.80%), Terrien marginal degeneration (3/501, 0.60%), and post-PRK ectasia (1/501, 0.20%). No cases of infectious keratitis were observed. Peripheral sterile infiltrates occurred in ten cases (10/501, 2.00%), all of which reacted well to topical steroids. Delayed epithelialization of more than seven days occurred in 14/501 (2.79%) patients, with all corneas showing full epithelialization after 12 days (Additional file 1: Table S1).

## Discussion

In this study of epi-off CXL performed in an office-based, non-sterile procedure room setting, adverse events were rare, with observed rates ranging from 0% to 2.79%. These rates are comparable to epi-off CXL complication rates reported in the literature. For example, in 2009, Koller et al. described a case series of 117 eyes with corneal ectasia that underwent Dresden protocol CXL (30 min of 3 mW/cm^2^ UV irradiation at 3 mW/cm^2^ intensity) [15]. Sterile infiltrates occurred in 9/117 (7.69%) of eyes and stromal scarring in three eyes (2.56%); no cases of infectious keratitis were observed. There have been several individual case reports describing infectious keratitis following epi-off CXL [16–20], but there are few data on larger patient groups, with the exception of Shetty et al. [21], who observed four cases of infectious keratitis amongst 2350 patients (0.17%). Serraro et al. reviewed the adverse event rates of epi-on and epi-off CXL procedures of 27 publications that comprised a total of 9397 eyes, 9006 of which were epi-off procedures [22]. In terms of epi-off procedures, infectious, bacteria, viral and herpetic keratitis rates were 2.26% (45/1990), 0.12% (2/1659), 0.62% (1/161) and 0.18% (4/2182), respectively. Corneal infiltrate rates were 2.0% (55/2776), and scarring occurred in 1.59% (49/3089). Reports by Dhawan et al. and Koppen et al. described four cases of infectious keratitis in 117 eyes (3.42%) undergoing epi-off Dresden protocol [23, 24].

While it is recognized that environmental heat and humidity can contribute to pathogen growth, and partially explain regional differences in rates and types of infectious keratitis, it is also reasonable to presume that these environmental factors could also influence post-procedural infection rates. However, given the strong pathogen-killing effects of CXL, rendering the cornea effectively “sterile” [5–7], the main drivers of post-procedural infection risk are not the method, setting, or environmental conditions that exist during the procedure. Rather, the drivers are in how carefully the cornea is handled after CXL is complete, highlighting the importance of patients carefully adhering to their post-procedural topical antimicrobial drug regimen and not rubbing their eyes [14].

This study has certain limitations. It is retrospective in nature and compares adverse event rates with those published in the literature, rather than having an operating room control group. During the period under consideration, the UV irradiation device and the riboflavin solution were changed. Even though the beam profiles were similar and UV output intensities were matched, the riboflavin solutions were similar in composition, both being hypo-osmolar, HPMC, and dextran-free. Moreover, different UV irradiation intensities and durations were applied, reflecting the evolution of clinical practice in CXL in Europe during this period.

The study included both thin (330 to < 400 µm) and ultra-thin (200 to < 330 µm) corneas treated with the sub400 protocol [25]. This protocol adapts the UV fluence delivered to patients' individual thinnest-point pachymetries to cross-link the cornea while maintaining an approximately 70 µm uncross-linked safety margin of basal stroma. This measure aims to protect the corneal endothelium from damage, as established by the Dresden protocol.

Most patients received 9 mW/cm^2^ UV intensity for 10 min. However, for certain groups of patients (predominantly pediatric) with particularly aggressive disease, the classic Dresden protocol (3 mW/cm^2^ for 30 min) [26] was applied for maximal corneal strengthening effect. The study being performed by a single surgeon, has the benefit of consistency and removing any variables that may be introduced by multiple surgeons, but may also limit the generalizability of the results. Finally, some procedures were performed with the patient sitting upright at the slit lamp to receive the UV irradiation, whereas other patients were irradiated lying supine. However, it has been shown that the position in which the patient receives UV irradiation does not materially influence the riboflavin distribution or depth of cross-linking effect [3, 27, 28].

It is worth comparing the adverse event rates of CXL with other ophthalmological procedures that were previously always performed in an operating room setting and are now increasingly being performed as office-based procedures. These include intravitreal injections (IVIs) of anti-VEGF drugs for the treatment of neovascular diseases of the retina [9–12] or cataract surgery [13], with the intention of making cost and resource savings [9–13]. Undoubtedly, injecting a substance into the vitreous cavity or performing intraocular surgery has the potential for serious infectious consequences. Nevertheless, published data show that IVI or cataract surgery performed in an office-based or examination room setting does not result in increased endophthalmitis rates [9–13]. For example, one meta-analysis of 1,275,815 IVIs found no difference in endophthalmitis rates between those performed in an office or an operating room setting [9]. Ianchulev et al. reported the results of a large single-center retrospective study of office-based cataract surgery (13,507 patients; 21,501 eyes), finding that “office-based efficacy outcomes were consistently excellent, with a safety profile expected of minimally invasive cataract procedures performed in ambulatory surgical centers and hospital outpatient departments” [13]. The safety of intraocular procedures and surgeries conducted in an office-based setting has been shown to be comparable to that of procedures performed in an operating room. In addition, the UV-riboflavin photochemical reaction inherent in CXL procedures is known to produce sufficient ROS to reduce the microbial load significantly [4]. This reduction in microbial load is so substantial that CXL can be successfully employed as a treatment for infectious keratitis [6], even as a stand-alone procedure [6, 29]. Given these established facts, the findings from our study lend further support to the concept of performing epi-off CXL safely in a procedure room. For the purposes of this discussion, a procedure room is defined as a room specifically designed and equipped for performing medical procedures. It is characterized by a ventilation system that ensures adequate airflow and minimizes the risk of infection, and thus makes it an acceptable alternative to an operating room.

Transitioning CXL from operating rooms to procedural rooms should significantly reduce costs, enhancing accessibility in low-to-middle income countries (LMICs) where financial barriers limit care. This shift has broad economic implications. Given the prevalence of vision loss due to corneal ectasias, early CXL intervention is crucial for vision preservation and prolonged economic productivity. This cost reduction and increased access could yield wider societal economic benefits, particularly in LMICs that have higher levels of currently unmet clinical need for CXL to treat corneal ectasias.

## Conclusion

The findings from this retrospective analysis of 501 epi-off CXL procedures indicate that there is no increase in the risk of postoperative infectious keratitis when performing epi-off CXL in a procedure room compared with operating room-based procedures previously published in the literature. This suggests that surgeons can be confident that epi-off CXL can be safely performed outside of the operating room setting. The fact that every CXL procedure reduces the microbial load on the cornea due to the UV-riboflavin photochemical reaction [5–7], and has been shown to be effective enough to be used as a monotherapy for the treatment of bacterial and fungal infectious keratitis, is also reassuring.

Furthermore, as transepithelial procedures have improved in their efficacy and are becoming more commonly performed [30], the more widespread adoption of CXL that spares the corneal epithelium should further reduce the risk of corneal infections during or after CXL, providing further reassurance that office-based CXL approaches can be performed as safely as CXL in an operating room.

### Supplementary Information

**Additional file 1: Table S1.** Adverse events narrative.

## Data Availability

Data are available from the corresponding author upon reasonable request.

## Abbreviations

BSS: Balanced salt solution
CXL: Corneal cross-linking
HPMC: Hydroxypropyl methylcellulose
IRB: Institutional review board
ROS: Reactive oxygen species
UV: Ultraviolet
VEGF: Vascular endothelial growth factor
