# Correction: Rates of infectious keratitis and other ocular surface adverse events in corneal cross-linking for keratoconus and corneal ectasias performed in an office-based setting: a retrospective cohort study

**DOI:** 10.1186/s40662-023-00360-3

**Published:** 2023-09-25

**Authors:** Farhad Hafezi, Emilio A. Torres-Netto, Leonard Kollros, Nan-Ji Lu, Nikki Hafezi, Cosimo Mazzotta, M. Enes Aydemir, Mark Hillen

**Affiliations:** 1 Laboratory for Ocular Cell Biology, Center for Applied Biotechnology and Molecular Medicine, University of Zurich, Zurich, Switzerland; 2 ELZA Institute, Dietikon, Switzerland; 3 USC Roski Eye Institute, University of Southern California, Los Angeles, CA USA; 4 Faculty of Medicine, University of Geneva, Geneva, Switzerland; 5 Deptartment of Ophthalmology, Wenzhou Medical University, Wenzhou, China; 6 Departmental Ophthalmology Unit, Alta Val d’Elsa Hospital, AUSL Tuscany South-East, Siena, Italy; 7 Postgraduate Ophthalmology School, University of Siena, Siena, Italy; 8 Siena Crosslinking Center, Monteriggioni, Siena, Italy

**Correction: Eye and Vision (2023) 10:36** **https://doi.org/10.1186/s40662-023-00354-1**

After publication of this article [1], it was brought to our attention that the second author's name "Emilio de Almeida Torres-Netto" should be corrected to "Emilio A. Torres-Netto".

The original publication [1] has been corrected.
