# Supplementary material for: Rates of infectious keratitis and other ocular surface adverse events in corneal cross-linking for keratoconus and corneal ectasias performed in an office-based setting: a retrospective cohort study
Source: Eye Vis (Lond). 2023 Sep 1;10:36. doi: 10.1186/s40662-023-00354-1 (PMC10472555; doi:10.1186/s40662-023-00354-1)
Supplement: Supplementary file 1 — Additional file 1: Table S1. Adverse events narrative. [file 40662_2023_354_MOESM1_ESM.docx]

**Table S1.** Adverse events narrative.

| **Indication** | **Gender** | **Age at procedure (years)** | **Pre-op pachymetry (µm)** | **Riboflavin** | **Riboflavin soaking time (mins)** | **Abrasion setting** | **Abrasion method** | **CXL device** | **UV intensity (mW/cm**²**)** | **UV duration (mm:ss)** | **Duration of defect (days)** |
| --- | --- | --- | --- | --- | --- | --- | --- | --- | --- | --- | --- |
| **Delayed re-epithelialization** | | | | | | | | |  |  |  |
| Keratoconus | Male | 35.7 | 350 | Ricrolin+ | 20 | Supine, microscope | Hockey knife | CXL-365 Vario | 3 | 9:00 | 8 |
| Keratoconus | Male | 39.7 | 486 | Ribo-Ker | 10 | Supine, microscope | Ethanol | C-Eye | 9 | 10:00 | 8 |
| Keratoconus | Male | 39.8 | 494 | Ricrolin+ | 10 | Supine, microscope | Ethanol | C-Eye | 9 | 10:00 | 8 |
| Keratoconus | Male | 17.4 | 466 | Ribo-Ker | 10 | Supine, microscope | Ethanol | C-Eye | 18 | 9:15 | 8 |
| Keratoconus | Male | 36 | 461 | Ribo-Ker | 10 | Supine, microscope | Ethanol | C-Eye | 9 | 10:00 | 9 |
| Keratoconus | Male | 19.7 | 517 | Ricrolin+ | 20 | Supine, microscope | Ethanol | CXL-365 Vario | 9 | 13:00 | 11 |
| Post-LASIK ectasia | Female | 53.3 | 414 | Ribo-Ker | 20 | Supine, microscope | Ethanol | CXL-365 Vario | 9 | 10:00 | 11 |
| Keratoconus | Male | 35 | 483 | Ricrolin+ | 20 | Supine, microscope | Hockey knife | CXL-365 Vario | 9 | 10:00 | 8 |
| Keratoconus | Male | 35.9 | 357 | Ricrolin+ | 20 | Supine, microscope | Hockey knife | CXL-365 Vario | 3 | 6:00 | 8 |
| Keratoconus | Male | 43.1 | 505 | Ribo-Ker | 10 | Supine, microscope | Ethanol | C-Eye | 18 | 9:15 | 8 |
| Keratoconus | Male | 26.6 | 333 | Ribo-Ker | 10 | Supine, microscope | Ethanol | C-Eye | 9 | 7:24 | 8 |
| Keratoconus | Male | 27.2 | 323 | Ribo-Ker | 10 | Supine, microscope | Ethanol | C-Eye | 9 | 7:24 | 8 |
| Keratoconus | Female | 26.2 | 470 | Ricrolin+ | 20 | Supine, microscope | Hockey knife | CXL-365 Vario | 3 | 16:00 | 12 |
| Keratoconus | Male | 32.3 | 373 | Ricrolin+ | 20 | Supine, microscope | Hockey knife | CXL-365 Vario | 3 | 8:00 | 9 |
| **Peripheral sterile infiltrates** | | | | | | | | |  |  |  |
| Keratoconus | Male | 27.9 | 371 | Ricrolin+ | 20 | Slit lamp | Hockey knife | CXL-365 Vario | 9 | 9:00 |  |
| Keratoconus | Female | 24 | 409 | Ricrolin+ | 20 | Supine, microscope | Amoils brush | CXL-365 Vario | 9 | 10:00 |  |
| Keratoconus | Male | 24.5 | 359 | Ricrolin+ | 20 | Supine, microscope | Hockey knife | CXL-365 Vario | 3 | 15:00 |  |
| Keratoconus | Female | 37.7 | 441 | Ricrolin+ | 20 | Supine, microscope | Hockey knife | CXL-365 Vario | 9 | 10:00 |  |
| Terrien marginal degeneration | Male | 25.5 | 573 | Ricrolin+ | 20 | Supine, microscope | Hockey knife | CXL-365 Vario | 3 | 30:00 |  |
| Keratoconus | Male | 22.9 | 456 | Ricrolin+ | 20 | Supine, microscope | Hockey knife | CXL-365 Vario | 3 | 30:00 |  |
| Pellucid marginal degeneration | Male | 51 | 485 | Ricrolin+ | 20 | Supine, microscope | Hockey knife | CXL-365 Vario | 9 | 10:00 |  |
| Keratoconus | Male | 35.8 | 390 | Ricrolin+ | 20 | Supine, microscope | Hockey knife | CXL-365 Vario | 3 | 18:00 |  |
| Keratoconus | Male | 55.4 | 471 | Ricrolin+ | 20 | Supine, microscope | Hockey knife | CXL-365 Vario | 9 | 10:00 |  |
| Keratoconus | Male | 29.5 | 516 | Ricrolin+ | 20 | Supine, microscope | Hockey knife | CXL-365 Vario | 9 | 10:00 |  |

SD = standard deviation; LASIK = laser in situ keratomileusis; UV = ultraviolet; CXL = corneal collagen cross-linking
